# Sudden cardiac death due to β_2_-agonist therapy: is a genetic basis overlooked?

**Published:** 2012-03

**Authors:** K Yalta, N Sivri, B Geyik, Y Aksoy, E Yetkin

**Affiliations:** Cardiology Department, Trakya Üniversity, Edirne, Turkey; Cardiology Department, Trakya Üniversity, Edirne, Turkey; Cardiology Department, Trakya Üniversity, Edirne, Turkey; Cardiology Department, Trakya Üniversity, Edirne, Turkey; Cardiology Department, IMC Hospital, Mersin, Turkey

β_2_-adrenoceptor agonists have long been widely used for the management of certain conditions, including chronic obstructive pulmonary disease (COPD). However, in recent years, sudden cardiac death (SCD) associated with the use of β_2_-adrenoceptor agonists has raised significant concerns about the safety profile of these agents. These drugs may have the potential to induce SCD in a proportion of susceptible subjects with structural heart diseases (SHD) including cardiomyopathies, and should not be routinely prescribed before ruling out these pathologies.^1^

A case of SCD with an undiagnosed cardiomyopathy, possibly associated with the administration of a β_2_-adrenoceptor agonist (for a bronchial asthma attack) has recently been reported, suggesting the presence of SHD as the most important determinant of SCD due to β_2_-agonist therapy.^1^ However, as described below, SCD due to β_2_-agonist therapy may also be closely associated with individual genetic susceptibility, particularly in subjects with apparently normal hearts, suggesting the need for thorough investigation of all candidates of β_2_-agonist therapy, with regard to clinical clues to an electrophysiological genetic basis for β_2_-agonist-induced SCD, before prescribing these drugs.

Genetically determined arrhythmogenic entities including ion channelopathies and catecholaminergic ventricular tachycardia (VT) have been regarded as important aetiologies of SCD, particularly in young victims^2^ with apparently normal hearts. Ion channelopathies may be overt or obscure in terms of resting ECG signs (with or without QT-interval prolongation) and generally present with a spectrum of clinical symptoms (syncopal attacks, SCD) that may be triggered by a variety of internal or external factors, including QT-interval-prolonging drugs.^2^

Previously, we reported a case of torsades de pointes (TdP) with a severely prolonged corrected QT (QTc) interval induced by an initial low-dose sotalol intake in the presence of a normal basal QTc interval, suggesting an individual genetic susceptibility to drug-induced pro-arrhythmia.^3^ Besides eliciting proclivity for malign arrhythmias in the presence of structural heart diseases and triggering acute coronary syndromes (ACS) in susceptible subjects, β-receptor stimulation is also well known to prolong QT interval. Therefore, it may be suggested that TdP due to a severely prolonged QT interval may be propounded as one of the fundamental mechanisms of β_2_-agonist-related SCD in subjects with a genetic basis for drug-induced pro-arrhythmia. Consistent with this notion, in a retrospective study comprising a large population of patients with long-QT syndrome (LQTS), β_2_-agonist therapy was found to be associated with an increased risk of cardiac events among these patients.^4^

However, the genetic basis of drug-induced SCD may not be solely attributable to overt or obscure ion channelopathy eliciting severe QT prolongation and TdP in response to certain agents. Impaired repolarisation heterogeneity manifesting as robust increases in a variety of indices, including T-wave alternance (TWA) and QT dispersion,^5^ and reduction in effective refractory period (ERP) in myocardial tissue in response to certain agents may also be associated with drug-induced pro-arrhythmias, suggesting another potential mechanism of β_2_-agonist-related SCD. In a previous study, inhaled fenoterol (a β_2_-agonist) was found to induce significant increases in QTc interval and QTc dispersion in a group of healthy volunteers.^6^ Interestingly, in this study, a proportion of subjects had much higher values of QTc dispersion after inhaled fenoterol administration, suggesting an individual oversensitivity to the effects of beta-agonists, including fenoterol, in certain subjects.^6^ Repolarisation heterogeneity in response to certain agents was previously suggested to demonstrate inter-individual variety,^5^ and hence may potentially harbour a genetic basis.

Besides excluding structural heart diseases in candidates of β_2_-agonist therapy, clinicians should also investigate a variety of risk factors, including specific symptomatology (palpitation, syncope), subtle or overt ECG findings (QTc-interval prolongation,^7^ etc) and history or family history of SCD (particularly in subjects with normal hearts) that are suggestive of an existing electrophysiological genetic basis for drug-induced SCD. On the other hand, it is well known that expertise in cardiology is not a primary skill of most chest physicians.7 However, in clinical practice, β_2_-agonists are usually prescribed by clinicians, including chest physicians, who are not familiar with the concept of drug-induced pro-arrhythmia. Thereby, clinical clues to an electrophysiological genetic basis for β_2_-agonist-induced SCD may easily be overlooked in the clinical setting, indicating the necessity of multi-disciplinary evaluation of certain patients before prescribing these agents.

Pharmacological challenge with inhaled β-agonists has been suggested to identify high-risk candidates of β-agonist therapy by monitoring potential dynamic alterations in the duration of QTc interval.^7^ Partially consistent with this recommendation, in the event of a strong suggestion of genetic basis (not all candidates),^4^ pharmacological challenge with β_2_-agonists should be performed in the hospital setting to demonstrate possible life-threatening increases in the QTc interval or indices of repolarisation heterogeneity in the candidates of β_2_-agonist therapy. Hence the potential risk of β_2_-agonist-induced pro-arrhythmia may be predicted, to some degree.

In conclusion, it may be suggested that even though β_2_-agonist-induced SCD is more likely to occur in the setting of structural heart diseases, a proportion of patients suffering SCD due to β_2_-agonist therapy may have apparently normal hearts (in post-mortem examination), indicating an electrophysiological genetic basis for β_2_-agonist-induced pro-arrhythmia in these patients. However, clinical clues to a genetic basis associated with β_2_-agonist-induced SCD may easily be overlooked in the clinical setting.

Therefore, besides excluding structural heart diseases before prescribing these agents, it is of utmost clinical importance to investigate the risk factors associated with a potential electrophysiological genetic basis, including specific symptomatology, subtle ECG findings, family history of SCD in candidates of β_2_-agonist therapy, through a multi-disciplinary approach in certain conditions. In selected cases, challenge with β_2_-agonists in the hospital setting may uncover a variety of genetically determined, obscure ECG findings (robust increases in QT dispersion), and hence may help identify patients at high risk for β_2_-agonist-related SCD, indicating avoidance of these agents in certain patients, even with apparently normal hearts.
